# Antibody microarray analysis of cell surface antigens on CD4+ and CD8+ T cells from HIV+ individuals correlates with disease stages

**DOI:** 10.1186/1742-4690-4-83

**Published:** 2007-11-26

**Authors:** Jing Qin Wu, Bin Wang, Larissa Belov, Jeremy Chrisp, Jenny Learmont, Wayne B Dyer, John Zaunders, Anthony L Cunningham, Dominic E Dwyer, Nitin K Saksena

**Affiliations:** 1 Retroviral Genetics Division, Center for Virus Research, Westmead Millennium Institute, Darcy Road, Westmead, NSW 2145, Sydney, Australia; 2 Medsaic Pty Ltd, Suite 145, National Innovation Centre; Australian Technology Park, Garden Street, Eveleigh, NSW 1430, Sydney, Australia; 3 Viral Immunology Laboratory, Australian Red Cross Blood Service, Clarence Street, NSW 2000, Sydney, Australia; 4 Center for Immunology, Darlinghurst, NSW, Sydney, Australia; 5 Department of Virology, ICPMR, CIDM Labs, Westmead Hospital, Westmead, NSW 2145, Sydney, Australia

## Abstract

**Background:**

Expression levels of cell surface antigens such as CD38 and HLA-DR are related to HIV disease stages. To date, the immunophenotyping of cell surface antigens relies on flow cytometry, allowing estimation of 3–6 markers at a time. The recently described DotScan antibody microarray technology enables the simultaneous analysis of a large number of cell surface antigens. This new technology provides new opportunities to identify novel differential markers expressed or co-expressed on CD4+ and CD8+ T cells, which could aid in defining the stage of evolution of HIV infection and the immune status of the patient.

**Results:**

Using this new technology, we compared cell surface antigen expression on purified CD4+ and CD8+ T cells between 3 HIV disease groups (long-term non-progressors controlling viremia naturally; HIV+ patients on highly active antiretroviral therapy (HAART) with HIV plasma viral loads <50 copies/ml; and HIV+ patients with viremia during HAART) and uninfected controls. Pairwise comparisons identified 17 statistically differential cell surface antigens including 5 novel ones (CD212b1, CD218a, CD183, CD3 epsilon and CD9), not previously reported. Notably, changes in activation marker expression were more pronounced in CD8+ T cells, whereas changes in the expression of cell membrane receptors for cytokines and chemokines were more pronounced in CD4+ T cells.

**Conclusion:**

Our study not only confirmed cell surface antigens previously reported to be related to HIV disease stages, but also identified 5 novel ones. Of these five, three markers point to major changes in responsiveness to certain cytokines, which are involved in Th1 responses. For the first time our study shows how density of cell surface antigens could be efficiently exploited in an array manner in relation to HIV disease stages. This new platform of identifying disease markers can be further extended to study other diseases.

## Background

HIV infection leads to characteristic alterations in the subset composition of circulating CD4+ and CD8+ T lymphocytes. The activation marker CD38, in particular, and its level of expression on CD8+ T cells is a marker that is strongly associated with immune activation, particularly during primary HIV-1 infection and progression to AIDS, respectively [1-3]. Furthermore, decreased expression of CD38 on CD8+ T cells is highly correlated with the effectiveness of antiretroviral therapy [4-8] and lack of activation and expression of CD38 and HLA-DR on CD4+ T cells correlates with long-term non-progression [9]. The enumeration of CD4+ T-lymphocytes by flow cytometry is used routinely in the clinical management of HIV-infected individuals to monitor the severity of immunodeficiency caused by HIV, and this acts as a basis for commencing HAART and prophylaxis for *Pneumocystis carinii *pneumonia [10]. However, more information on the progression to immunodeficiency may be found in the detailed subset composition of CD4+ and CD8+ T cells, but this is currently restricted to research studies.

To date, the immunophenotyping of CD antigens relies on flow cytometry. Although very reliable, the flow cytometry only allows estimation of 3–6 markers in a given assay. The recently developed antibody microarray technology enables the simultaneous analysis of a large number of cell surface antigens on a single chip. This new technology may permit the identification of novel differential markers expressed or co-expressed on CD4+ and CD8+ T cells, which could aid in defining the stage of evolution of HIV infection and the immune status of the patient [11]. This antibody microarray also has significant advantages over gene expression microarray because it profiles cells at the level of protein expression, rather than relying on quantifying mRNA expression levels. The power of this technology as an adjunct to flow cytometry was recently highlighted by Woolfson *et al*. [12], who used a similar antibody microarray to demonstrate the conservation of unique cell surface antigen mosaics in cryopreserved PBMCs from HIV+ individuals.

Here, we have used an antibody microarray constructed on the surface of a nitrocellulose coated slide to simultaneously analyze 135 different cell surface antigens (128 cluster of differentiation antigens plus 7 other surface antigens) on peripheral blood CD4+ and CD8+ T cell subsets from HIV+ and HIV- individuals. A comparison of CD4+ and CD8+ T cells purified from peripheral blood of three different HIV-infected patient groups (Table 1, HIV+ therapy naïve long-term non-progressors with high CD4+ and CD8+ T cell counts; HIV+ patients on HAART with plasma viremia below detectable levels; and viremic patients on HAART) and HIV seronegative individuals showed 17 statistically differential cell surface antigens, 5 of which were novel. Furthermore, we demonstrate that changes in the expression of activation markers were more pronounced in CD8+ T cells, whereas changes in the expression of cell membrane receptors for cytokines and chemokines were more pronounced in CD4+ T cells.

**Table 1:** Patient clinical details of viral load, CD4+ and CD8+ T cell counts at the time of sample collection^a^

**Patient**	**Date**	**Age**	**CD4 counts (cells/μl)**	**CD8 counts (cells/μl)**	**Viral Load (copies/ml)**	**Disease Group**
1	20/06/06	55	380	790	<50	BDL
2	21/06/06	23	355	492	<50	
3	27/06/06	61	691	1538	<50	
4	27/06/06	42	425	721	<50	
5	02/08/06	62	1037	607	<50	
6	02/08/06	52	750	368	<50	
7	23/08/06	52	799	449	<50	
8	03/09/06	49	371	1818	<50	
9	12/09/06	62	458	206	<50	
10	13/09/06	39	901	870	<50	
11	20/09/06	61	782	1936	<50	
12	02/08/06	54	292	2989	352	VIR
13	21/06/06	38	560	980	49500	
14	16/08/06	39	500	2064	32600	
15	15/08/06	57	461	2306	46900	
16	29/08/06	41	390	463	567	
17	07/09/06	61	170	529	345	
18	12/09/06	43	251	642	1700	
19	15/09/06	50	265	170	105	
20	20/09/06	38	231	1289	46900	
21	03/10/06	45	286	1144	388	
22	28/06/06	58	690	650	<50	LTNP
23	28/06/06	50	817	513	<50	
24	18/07/06	78	880	860	<50	
25	21/07/06	58	760	1800	<50	
26	03/10/06	32	1121	790	128	

^a ^Plasma viral load was measured using the Quantiplex HIV RNA3.0 (Chiron bDNA) assay with a lower limit of detection of 50 HIV-1 copies/ml (Chiron Diagnostics, Halstead, United Kingdom).

## Results

The pairwise comparisons of groups identified 17 statistically differential cell surface antigens, of which, CD212b1, CD218a, CD183, CD3epsilon and CD9 have not been previously reported in the context of HIV disease.

### Discriminatory antibodies for CD4+ T cells

**Figure 1:**
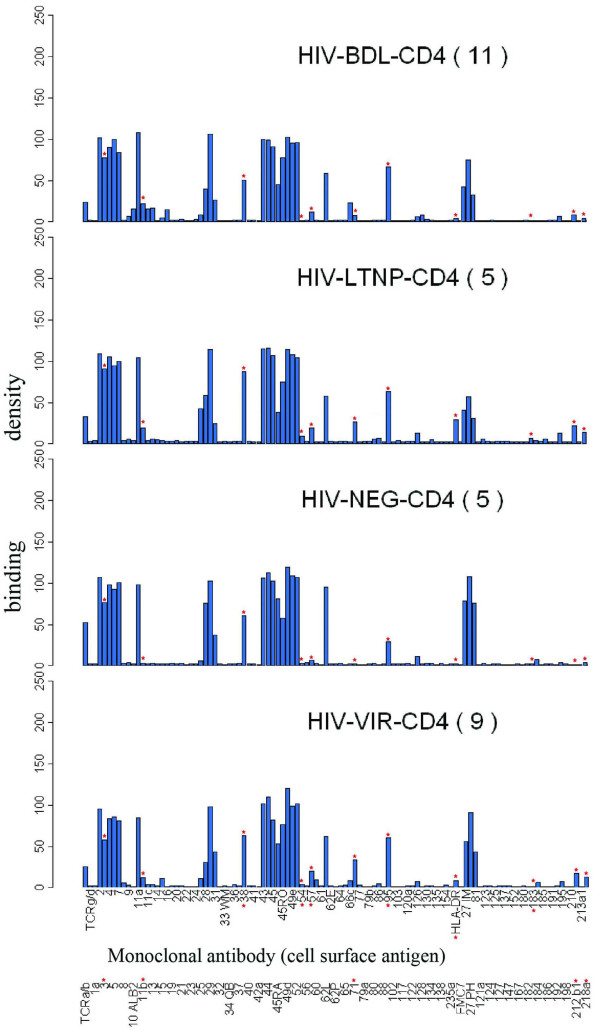
Antibody response charts of CD4+ T cells. The bar charts represent the average immunophenotypes, or signatures, for each of the disease categories. Asterisks show the antigens which were significantly up-or down-regulated in paired comparisons of the disease groups. Labeling on the x-axis refers to monoclonal antibodies and their specificities against the corresponding antigens and the y-axis the binding densities.

**Figure 2:**
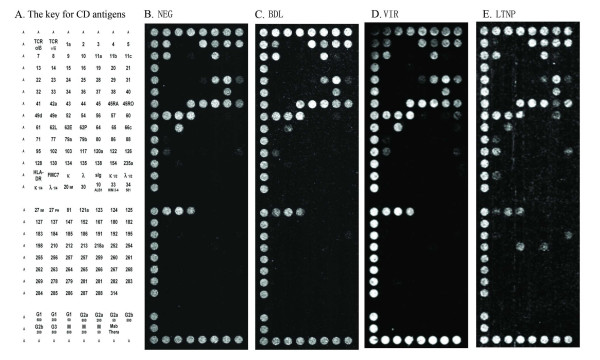
Composite dot scan patterns of antibody binding for CD4+ T cells. Half of a duplicate array was shown with the alignment dots "A" at left, top and bottom. Alignment dots are a mixture of CD44 and CD29 antibodies. (A) The key for CD antigens on the DotScan array, (B) NEG, (C) BDL, (D) VIR and (E) LTNP. Binding patterns shown here are representatives from each group. They may not fully reflect all the significant antigens from statistical analysis because of individual variability in antigen expression.

The signature pattern for each group and dot pattern from which the raw data were derived is shown in Figure 1 and 2, respectively. In pairwise comparisons, CD4+ cells from the HIV+ individuals differed from those of the NEG group, as shown by the significant upregulation of CD71, CD212b1, HLA-DR, CD95, CD57 and CD11b on the CD4+ cells of one or more of the HIV+ groups, as shown in Table 2. Although several other antigens showed increased (CD218a and CD86 in VIR) or decreased (CD27 in LTNP, CD45RA in BDL and CD28 in VIR) expression compared to the NEG group, these did not always reach statistical significance, with p values ranging from 0.0540 to 0.0744.

**Table 2:** Discriminatory antibodies for CD4+ T cells^a^

**Discriminatory Antibody**	**Up(+)/Down(-)**	**P Value**
**BDL vs. NEG**		
*CD11b*	+	*0.0599*
*CD95*	+	*0.0619*
*CD45RA*	-	*0.0679*
**LTNP vs. NEG**		
CD71	+	0.0151
CD 212 b1	+	0.0307
HLA-DR	+	0.0312
*CD27 PH*	-	*0.0560*
*CD11b*	+	*0.0589*
*CD95*	+	*0.0752*
**VIR vs. NEG**		
CD71	+	0.0032
CD11b	+	0.0227
CD57	+	0.0307
CD95	+	0.0349
*CD28*	-	*0.0540*
*CD218a*	+	*0.0732*
*CD86*	+	*0.0744*
**BDL vs. LTNP**		
CD183	-	0.0105
HLA-DR	-	0.0270
CD38	-	0.0316
CD71	-	0.0385
CD218a	-	0.0470
*CD212 b1*	-	*0.0687*
*CD54*	-	*0.0695*
**VIR vs. LTNP**		
CD183	-	0.0451
CD3epsilon	-	0.0489
*CD27 PH*	+	*0.0829*
**VIR vs. BDL**		
CD71	+	0.0195
CD218a	+	0.0355
CD54	+	0.0421

^a^Six paired comparisons of CD markers on CD4+ cells providing significant discrimination between patient groups, with relative changes in CD antigen binding in the former vs. the latter denoted by "+" and "-" to indicate increase and decrease, respectively. Antigens that did not achieve statistical significance (p > 0.05) but have been previously reported in HIV disease context or have been found to be significant in other pair comparisons are also shown in italic. The "PH" in CD27 PH distinguishes this Pharmingen antibody (clone M-T271) from the Immunotech antibody 27 IM (clone 1A4-CD27).

Average CD11b and CD95 expression also increased in all 3 HIV+ groups compared to NEG individuals, but this upregulation reached significance only in VIR, the p value in BDL and LTNP ranging from 0.0589 to 0.0752. Variability in antigen expression within the latter groups may contribute to this lack of significance, though the differences may reach significance with increased group sizes.

CD71, CD218a and CD54 were upregulated in both the LTNP and the VIR groups compared with BDL, with p values of < 0.05 for all except CD54 in the LTNP-BDL comparison (p = 0.0695). LTNP-CD4 differed from BDL and/or VIR groups in that CD71, HLA-DR, CD38, CD3epsilon and CD183 were significantly upregulated.

### Discriminatory antibodies for CD8+ T cells

**Figure 3:**
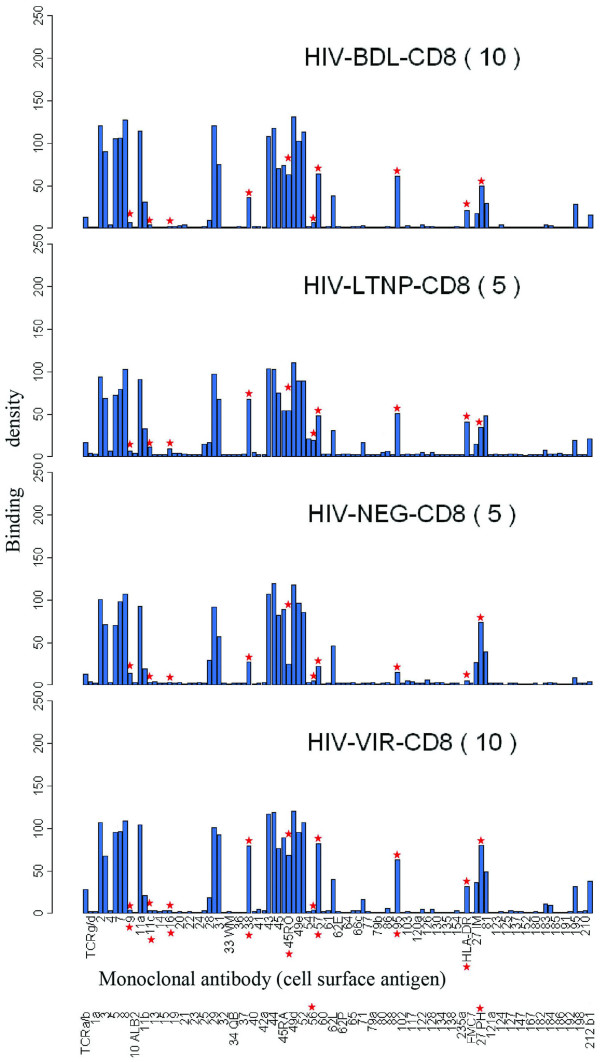
Antibody response charts of CD8+ T cells. The bar charts represent the average immunophenotypes, or signatures, for each of the disease categories. Asterisks show the antigens which were significantly up-or down-regulated in paired comparisons of the disease groups. Labeling on the x-axis refers to monoclonal antibodies and their specificities against the corresponding antigens and the y-axis the binding densities.

**Figure 4:**
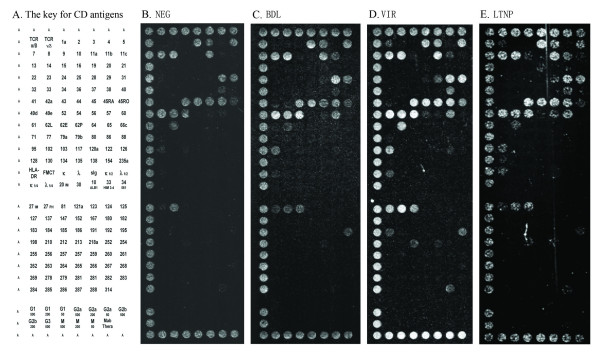
Composite dot scan patterns of antibody binding for CD8+ T cells. Half of a duplicate array was shown with the alignment dots "A" at left, top and bottom. Alignment dots are a mixture of CD44 and CD29 antibodies. (A) The key for CD antigens on the DotScan array, (B) NEG, (C) BDL, (D) VIR and (E) LTNP. Binding patterns shown here are representatives from each group. They may not fully reflect all the significant antigens from statistical analysis because of individual variability in antigen expression.

The signature pattern for each group and representative dot pattern from which the raw data were derived are shown in Figure 3 and 4, respectively. In pairwise comparisons, CD8+ cells from HIV+ individuals differed from the NEG group, as shown by the significant upregulation of HLA-DR, CD57, CD11c, CD45RO and CD95 in one or more of the HIV+ groups, with all 3 HIV+ groups showing an increase in average HLA-DR, CD57, CD45RO and CD95 expression (Table 3). There was significant downregulation of CD9 (in VIR) and CD27 (in LTNP), while increases in CD212b1 (in LTNP and VIR) did not reach significance (p = 0.0745–0.0776). CD38 was significantly upregulated in the VIR group compared with BDL and NEG groups. LTNP differed from BDL and VIR groups in that CD8+ cells showed higher expression of CD11c, CD16 and CD56, all differences being statistically significant except CD56 in the LTNP-BDL comparison (p = 0.0871).

**Table 3:** Discriminatory antibodies for CD8+ T cells^a^

**Discriminatory Antibody**	**Up(+)/Down(-)**	**P Value**
**BDL vs. NEG**		
CD57	+	0.0272
CD45RO	+	0.0307
HLA-DR	+	0.0315
*CD95*	+	*0.0538*
**LTNP vs. NEG**		
HLA-DR	+	0.0039
CD11c	+	0.0072
CD38	+	0.0219
CD27 PH	-	0.0310
*CD95*	+	*0.0545*
*CD57*	+	*0.0591*
*CD16*	+	*0.0631*
*CD212b1*	+	*0.0745*
*CD45RO*	+	*0.0953*
**VIR vs. NEG**		
CD57	+	0.0006
CD45RO	+	0.0067
CD38	+	0.0091
CD95	+	0.0091
HLA-DR	+	0.0190
CD9	-	0.0268
*CD212b1*	+	*0.0776*
**BDL vs. LTNP**		
CD11c	-	0.0049
CD16	-	0.0093
*CD56*	-	*0.0871*
**VIR vs. LTNP**		
CD11c	-	0.0017
CD56	-	0.0121
CD16	-	0.0308
*CD27PH*	+	*0.0585*
*CD9*	-	*0.0862*
**VIR vs. BDL**		
CD38	+	0.0124

^a^Six paired comparisons of CD markers on CD8+ cells providing significant discrimination between patient groups, with relative changes in CD antigen binding in the former vs. the latter denoted by "+" and "-" to indicate increase and decrease, respectively. Antigens that did not achieve statistical significance (p > 0.05) but have been previously reported in HIV disease context or have been found to be significant in other pair comparisons are also shown in italic.

## Discussion

To date the analysis of cell surface antigens on peripheral blood lymphocytes of HIV infected individuals has been largely carried out with whole PBMC using flow cytometry. In this study we used DotScan antibody microarray technology (Medsaic Pty. Ltd, Sydney, Australia) to simultaneously analyze CD4+ and CD8+ T cell subsets obtained from HIV+ individuals at different stages of HIV disease for the expression of 135 different cell surface antigens. For the first time, our study shows how the immunophenotype of diverse blood cell types (in this case CD4+ and CD8+ T cells) can be exploited to study various HIV disease stages using antibody microarray technology. Our analyses identified 17 statistically significant (p < 0.05) differentiating CD antigens distributed between CD4+ and CD8+ T cells. Of these, 11 (CD71, CD212b1, HLA-DR, CD57, CD95, CD11b, CD38, CD3epsilon, CD218a, CD54 and CD183) were differential for CD4+ T cells, while 10 (CD9, CD11c, CD16, CD38, CD27, CD45RO, CD56, CD57, CD95 and HLA-DR) for CD8+ T cells. Among these, CD212b1, CD218a, CD183, CD3epsilon and CD9 have not been previously described in relation to HIV disease.

For the activation markers, the results are in accordance with previous studies using flow cytometry, confirming the utility of this new technology. As previously reported [13,14], HLA-DR and CD38 were significantly upregulated on both CD4+ and CD8+ T cells during HIV infection. On CD4+ T cells, we observed significant upregulation of HLA-DR in LTNP comparison to BDL and NEG groups, suggesting that the level of activation of CD4+ T cells from the BDL group was reduced by HAART to a level similar to the NEG group. For CD38, a significant increase was detected in the LTNP compared to the BDL group. This may be due to the intermediate expression of CD38 on naïve CD4+ T cells, but this was not confirmed as this study did not differentiate between naïve and memory CD4+ T cells. On CD8+ T cells, significant upregulation of HLA-DR was observed in all HIV+ groups, while CD38 upregulation was seen in two HIV+ groups (LTNP and VIR compared to the NEG group) and the pairwise comparison of VIR versus BDL group, which is in full accordance with previous studies showing CD38 expression on CD8+ T cells is a marker associated with HIV disease progression [1,2]. The increased expression of CD45RO on CD8+ T cells during HIV infection has also been well documented [15], supporting our observation that increases in CD45RO expression were significant, or close to significant, on CD8+ T cells in all 3 HIV+ groups. However, CD45RO modulation was not observed on CD4+ T cells, confirming flow cytometry data [13], which also showed that the proportion of CD4+ T cells expressing CD45RO remained relatively unchanged. Taken together, it appears that for the activation markers mentioned above, significant changes in expression were more pronounced on CD8+ than CD4+ T cells.

In addition, CD27 was significantly downregulated on CD8+ T cells in the LTNP group compared to the NEG group. It has been suggested in one study that HIV-specific CD8+ T cells that have differentiated to the CD27- stage are related to the delayed disease progression [16], while another study has also observed a similar trend [17]. The CD8+ T cells used in our study were not selected for HIV-specificity, and hence the LTNP status appears to be related to a general downregulation of CD27.

We also observed significantly increased CD95 expression on both CD4+ and CD8+ T cells in the VIR group. Although partially elevated CD95 levels were also observed in BDL on HAART and LTNP groups, the increases were not significant, which is consistent with reduced immune activation in patients with reduced viral replication. Increased Fas-receptor (CD95) expression on CD4+ and CD8+ lymphocytes has previously been demonstrated in a large group of HIV-1-infected patients when compared against normal controls [18], and evidence also suggests that poor responders to antiretroviral therapy may have significantly higher CD95 expression [19]. These findings, together with our results, suggest that significantly increased CD95 expression may relate to antiretroviral therapy failure.

The most notable feature for the activation markers was significant downregulation of CD9 expression on CD8+ T cells in the VIR group compared to the NEG group. CD9 belongs to a transmembrane protein family known as the tetraspanin family. It has been proposed that these proteins act as scaffolding proteins by laterally organizing cellular membranes via specific associations with each other and distinct integrins. A recent study has shown that the tetraspanin-enriched microdomains on the cell membrane can function as gateways for HIV egress [20]. The overall functional relevance of this antigen in the context of HIV disease requires further investigation.

It has been suggested that CD57 is a marker for replicative senescence [21,22]. We found a significant increase of CD57 expression on CD8+ T cells in 2 of the 3 HIV infected groups (BDL and VIR) compared with NEG group, while on CD4+ T cells, only the VIR group showed significantly increased expression. For HIV-specific CD8+ cells, diminished proliferative capacity results in a memory T cell population with reduced capacity to control infections [23]. Although we did not study pure HIV-specific T cells, the significantly increased expression of CD57 seems to be directly related to disease progression. In contrast to CD57 which is linked to replicative senescence, the transferrin receptor CD71 has been reported as a marker for T cell proliferation [24]. DotScan analysis showed that CD4+ T cells of LTNP and VIR groups expressed significantly higher levels of expression of CD71 than that of the BDL group. Consistent with studies showing a significant increase in T cell turnover in HIV infection [25-27], the upregulation of CD71 expression in the LTNP group may indicate that the cells have recently cycled. On the other hand, the upregulation of both CD57 and CD71 in the VIR group may indicate the activation rather than the cell proliferation index [28]. Since CD71 constitutively cycles from endosomes to the cell surface and back again [29], the microarray was significantly better at detecting CD71 expression than flow cytometry.

Three cytokine receptors (CD183, CD218a, and CD212b1) were found to be significantly upregulated on CD4+ T cells in LTNP group, indicating a polarized Th1 cell immune response in LTNP group in HIV infection. The CD4+ T cells of the LTNP group expressed higher levels of CD183 (CXCR3) than those of the BDL and VIR groups. Since CD183 is reported to be preferentially expressed on Th1 versus Th2 cells in peripheral blood [30,31] and to be found on a high percentage of CD4+ T cells in type 1-dominated inflammatory processes [32], the upregulation of this protein in the LTNP group may imply a polarization towards a Th1 immune response in LTNP group. CD218a (α chain of IL18R) was significantly upregulated on the CD4+ T cells of LTNP and VIR groups compared with the BDL group. CD212b1 (beta1 chain of IL12R) was also significantly upregulated on the CD4+ T cells of the LTNP group compared to the NEG group. A previous study on the expression of cytokine receptors on lymphocytes from patients suffering from a disorder associated with raised Th1 cytokine production showed that the percentage of CD218a+ and CD212b1+ cells within the CD4+CD45RA+ subset is significantly higher in these patients than in healthy subjects [33]. By analogy, in HIV disease, the upregulation of CD218a and CD212b1 in HIV disease may also indicate a chronically polarized immune response towards Th1 in LTNP group. With regard to cytokine receptors, although previous studies have shown that the level of CD127 (IL-7R) expression represents a major difference between HIV+ subjects and controls [34-36], we did not observe any difference between groups. This is due to the lack of cell binding to CD127 antibody on the microarray, which may attribute to either low cell numbers of CD127+ or the low affinity of this antibody used.

With regard to cell signaling, CD3epsilon expression on CD4+ T cells was found to be significantly lower in the VIR group than in LTNP, which indicates that the expression level of CD3epsilon can be used to differentiate VIR and LTNP groups. A previous study on HIV+ patients has shown that the expression of CD3 complex (gamma, delta, epsilon) is downregulated on T cells compared to healthy control [37], while our study is the first to relate one of the components of CD3 complex, CD3epsilon, to HIV disease status, yet the biological significance of CD3epsilon expression in HIV disease requires further elucidation. Interestingly, the significant changes in expression of cell membrane receptors mentioned above (CD183, CD218a, CD212b1 and CD3epsilon) were all observed on CD4+ T cells, but not on CD8+ T cells.

Significant group-specific differences were also found for 3 cell adhesion molecules (CD11b, CD11c and CD54), which may have significant implications for the pathogenesis of HIV disease, since adhesion molecules can affect cell distribution, migration and immune response. CD11b was significantly upregulated on CD4+ T cells in the VIR group compared with the NEG group. This increase was also seen in the BDL and LTNP groups, but was not statistically significant. Although there is evidence for increases in CD8+/CD11b+ T cells during progression of HIV infection in asymptomatic patients [38], we are not aware of any previous reports of modulations in CD11b expression on CD4+ T cells of HIV+ patients. CD11c, which has mainly been studied in relation to dendritic cells in HIV disease, was found to be expressed at significantly higher levels on CD8+ T cells in the LTNP group than in the other 2 HIV+ groups. Although the upregulation of CD11c after HIV-1 infection has been reported at mRNA levels [39], it has not previously been documented at protein levels. CD54, also known as intercellular adhesion molecule-1, was significantly elevated on CD4+ T cells in VIR compared to the BDL group. This is consistent with a previous report of the involvement of intercellular adhesion molecule-1 in syncytia formation and virus infectivity and the increase in its expression on lymphocytes in HIV infection [40].

Interestingly, the expression levels of two NK associated receptors on CD8+ T cells, CD16 and CD56 were significantly higher for LTNP than for BDL and VIR groups, though not all detected differences reached statistical significance (p = 0.0093 to 0.0871). CD56 is expressed on a subset of CD8+ T cells (mature cytolytic effector cells) and it has previously been suggested that the defective expression of CD56 on these cells in HIV-infected individuals could contribute to the decreased peripheral blood T-cell cytotoxicity found in HIV infection [41]. These findings, together with ours, support the hypothesis that the CD8+ T cells from LNTP may have stronger cytotoxic activity than those from other HIV+ individuals. CD16 expression on CD8+ T cells in HIV disease has not previously been reported. However, increases in CD8 T cells expressing NK associated receptors have been reported in melanoma patients, and these cells display an effector phenotype [42]. Similar changes may also occur in HIV patients, and the implication of these changes needs further investigation.

## Conclusion

DotScan antibody microarray technology enabled the identification of 3 distinct HIV disease groups based on an extensive immunophenotypic characterization of the patients' CD4+ and CD8+ peripheral blood T cells. This research not only confirmed previously reported findings from flow cytometric investigations, but also demonstrated the power of the antibody microarray technology, by identifying 5 new cell surface antigens that may potentially be associated with HIV disease stages. Simultaneous screening for a large number of cell surface antigens revealed that changes in the expression of activation markers were more pronounced in CD8+ T cells, whereas changes in the expression of cell membrane receptors for cytokines and chemokines were more pronounced in CD4+ T cells.

Since these changes were shown to be related to the disease status, we suggest that the use of this technology will facilitate further investigation of the causes and control of HIV disease progression and eventually lead to a better understanding of the pathogenesis of the disease. Our study is the first to demonstrate how density of cell surface antigens can be efficiently exploited in an array manner in relation to disease stages. This new platform of identifying disease markers can be further extended to study other diseases. Increasing patient group size should correspondingly improve the statistical significance of the observed differences in antigen expression associated with disease stage. A simplified protocol of direct purification of CD4+ or CD8+ T cells from whole blood may also allow a broader diagnostic utility. Serial time course studies of patients during their disease progression should also provide useful information on the modulation of cell surface antigens over time and could potentially identify new prognostic and therapeutic markers relevant to HIV disease, enabling prediction of patient responsiveness to therapy.

## Methods

### Patient profiles

Blood (20 ml EDTA) was obtained from 26 HIV+ individuals attending HIV clinic at the Westmead Hospital (Table 1) and 5 HIV- healthy individuals from the Australian Red Cross, Sydney. This study has been approved by the Western Sydney Area Health Services and all blood samples were obtained upon written informed consent. The patient groups were: (**1**) Healthy HIV- individuals (NEG; n = 5); (**2**) HIV+ individuals on HAART with "below detectable levels" of plasma viremia and classed as patients controlling viremia with HAART (BDL; n = 11 for CD4; n = 10 for CD8; one CD8 sample was excluded as it failed to meet the internal control criteria); (**3**) HIV+ individuals on HAART with detectable plasma viremia (VIR; n = 9 for CD4; n = 10 for CD8; one CD4 sample was excluded as it failed to meet the internal control criteria); (**4**) Treatment naïve HIV+ long-term non-progressors (LTNP; n = 5), who have maintained high CD4+ T cell counts (>500 cells/μl), with the average infection time of >20 years and natural control of plasma viremia to below detectable levels. One of the 5 LTNP patients (patient 26) in this category did show very low plasma viremia (128 HIV RNA copies/ml of plasma), but was included because this patient met all the other selection criteria.

### Purification of CD4+ and CD8+ T cells

A single blood sample (20 ml) was obtained from each patient. After separation of plasma, PBMC were isolated by Ficoll-gradient centrifugation and then purified. CD4+ and CD8+ T cells, respectively, were obtained by positive isolation with antibody-conjugated magnetic beads according to the manufacturer's instructions (Dynal Biotech, Oslo, Norway). Flow-cytometric analysis performed on separated CD4+ and CD8+ T cell populations demonstrated that in CD4+ T cell isolations 99.2% ± 0.165% (mean ± SD) of cells were single positive for CD4 marker, while 99.1% ± 0.128 (mean ± SD) of purified CD8+ cells were single positive for CD8 marker [43]. Absence of binding of purified CD8+ T cells to the CD4 antibody and *vice versa *further confirmed that cross contamination was negligible and would not compromise assay specificity.

### CD antibody microarrays

Medsaic Pty. Ltd. (Eveleigh, NSW, Australia) provided the DotScan^TM ^microarrays, prepared as previously described [44]. Monoclonal antibodies were purchased from the following companies: Coulter and Immunotech from Beckman Coulter (Gladesville, NSW, Australia), Pharmingen (BD Biosciences, North Ryde, NSW, Australia), Biosource International (Applied Medical, Stafford City, QLD, Australia), Serotec (Australian Laboratory Services, Sydney, NSW, Australia), Sigma-Aldrich (Castle Hill, NSW, Australia), Biotrend, Biodesign and MBL (Jomar Diagnostics, Stepney, SA, Australia), Chemicon Australia (Boronia, VIC, Australia), Leinco Technologies (St. Louis, MO, USA) and Calbiochem (Merck, Kilsyth, VIC, Australia). Antibody solutions were reconstituted as recommended, and stored in aliquots with 0.1% (w/v) BSA at -80°C; Pharmingen antibodies were generally stored at 4°C. Antibodies were used for making microarrays at concentrations ranging from 50–1000 μg protein/ml.

### Immunophenotyping of ex vivo purified CD4+ and CD8+ T cells

Purified CD4+ and CD8+ T cell populations were tested on antibody microarrays using DotScan technology as previously described [45]. Briefly, a 300 μL aliquot of either purified CD4+ or CD8+ cell suspension (= 4 × 10^6 ^cells) was incubated for 30 min on the microarray chip, after which unbound cells were removed by gentle immersion in PBS. Captured cells were fixed and imaged using a Medsaic DotReader™ and dot intensities were quantified for each antigen in duplicate using Dotscan data analysis software on an 8-bit pixel grey scale from 0–255 that reflects the level of expression of a particular antigen as well as the proportion of cells expressing that antigen [45]. The limit of detection using the optical scanner is approximately 100 cells/antibody dot. Each microarray has alignment dots, which establishes the location of each dot on the array and also served as the internal control to measure the distribution of cells. The dot pattern obtained is the immunophenotype of that population of leukocytes.

The main strength of antibody microarray is its capacity to rapidly screen for a large number of antigens, producing an extensive immunophenotype using a relatively small number of cells in a single assay. However, it would not provide all of the information obtained by flow cytometry such as multiparameter analysis on single cells and level of antigen expression per cell. When the same sample is tested by the same operator on 3 different arrays (unpublished data), the coefficient of variation (CV) for binding densities tends to be low (8.3%) for dots of high density (>50 pixels), but higher (33.3%) for dots of low density (2–50 pixels). Reproducible dot binding patterns can be achieved if a technically consistent standard assay protocol is followed for all assays.

### Statistical derivations

The objective of this analysis was to identify antibody dots showing differential levels of binding of cells derived from different disease categories, where the sample categories had been established *a priori*. No fewer than 5 samples per group were included for statistical analysis, as required for the application of Medsaic's standard bioinformatics tools used in this study. Data were log transformed and log transformed antibody bindings have a symmetrical distribution which shows reasonable stability of variance with mean expression. Following transformation, the distributional properties for individual antibodies were examined using box plots and kernel density estimators. Differential expression was analysed on an antibody-by-antibody basis. Individual intra-group comparisons were first carried out, followed by pairwise inter-group comparisons between the four study groups. The "Antibody Ranking" analysis of the array binding results was carried out as a one way analysis of variance, which provided p values and adjusted p values for each antibody and ranked the antibodies in order of significance. P values were adjusted using Holm's method, a conservative approach to maintain strong control of an inflated type I error rate [46]. Differential expression of antigens was identified by paired comparisons of the 4 study groups, with differences reaching statistical significance when the adjusted p value was less than 0.05.

## Abbreviations

Abbreviations used in this paper: 

BDL, below detection level;

HAART, highly active antiretroviral therapy;   

LTNP, long-term non-progressor;

NEG, HIV seronegative individuals; 

VIR, viremic patients.

## Competing interests

The author(s) declare that they have no competing interests.

## Authors' contributions

JQW fully performed the work, analyzed data and wrote the paper. BW contributed to the writing. LB and JC analyzed data, did statistical evaluation, contributed to the technology and the writing. JL and DED contributed to vital patient samples and immunological interpretation of findings. WBD, JZ, ALC, NKS designed the research project, supervised this work and contributed to the writing. All authors read and approved the final manuscript.
